# *Cynanchum paniculatum* and Its Major Active Constituents for Inflammatory-Related Diseases: A Review of Traditional Use, Multiple Pathway Modulations, and Clinical Applications

**DOI:** 10.1155/2020/7259686

**Published:** 2020-07-22

**Authors:** Jing-xian Chen, Chien-shan Cheng, Jie Chen, Ling-ling Lv, Zi-jie Chen, Chuan Chen, Lan Zheng

**Affiliations:** ^1^Department of Traditional Chinese Medicine, Shanghai Jiao Tong University School of Medicine Affiliated Ruijin Hospital, Shanghai 200025, China; ^2^Workstation of Xia Xiang, National Master of Traditional Chinese Medicine, Department of Traditional Chinese Medicine, Ruijin Hospital, School of Medicine, Shanghai Jiao Tong University, Shanghai 200025, China; ^3^Department of Integrative Oncology, Fudan University Shanghai Cancer Center, Shanghai 200032, China; ^4^Department of Oncology, Shanghai Medical College, Fudan University, Shanghai 200032, China; ^5^Department of Orthopedics, Shanghai Institute of Traumatology and Orthopedics, Ruijin Hospital, Shanghai Jiao Tong University School of Medicine, Shanghai 200025, China; ^6^Shanghai Yangpu Hospital of Traditional Chinese Medicine, Shanghai 200090, China; ^7^Shanghai Geriatrics Institute of Chinese Medicine, Shanghai University of Traditional Chinese Medicine, Shanghai 200031, China

## Abstract

*Cynanchum paniculatum* Radix, known as *Xuchangqing* in Chinese, is commonly prescribed in Chinese Medicine (CM) for the treatment of various inflammatory diseases. The anti-inflammatory property of *Cynanchum paniculatum* can be traced from its wind-damp removing, collaterals' obstruction relieving, and toxins counteracting effects as folk medicine in CM. This paper systematically reviewed the research advancement of the pharmacological effects of *Cynanchum paniculatum* among a variety of human diseases, including diseases of the respiratory, circulatory, digestive, urogenital, hematopoietic, endocrine and metabolomic, neurological, skeletal, and rheumatological systems and malignant diseases. This review aims to link the long history of clinical applications of *Cynanchum paniculatum* in CM with recent biomedical investigations. The major bioactive chemical compositions of *Cynanchum paniculatum* and their associated action mechanism unveiled by biomedical investigations as well as the present clinical applications and future perspectives are discussed. The major focuses of this review are on the diverse mechanisms of *Cynanchum paniculatum* and the role of its active components in inflammatory diseases.

## 1. Introduction

**Figure 1:**
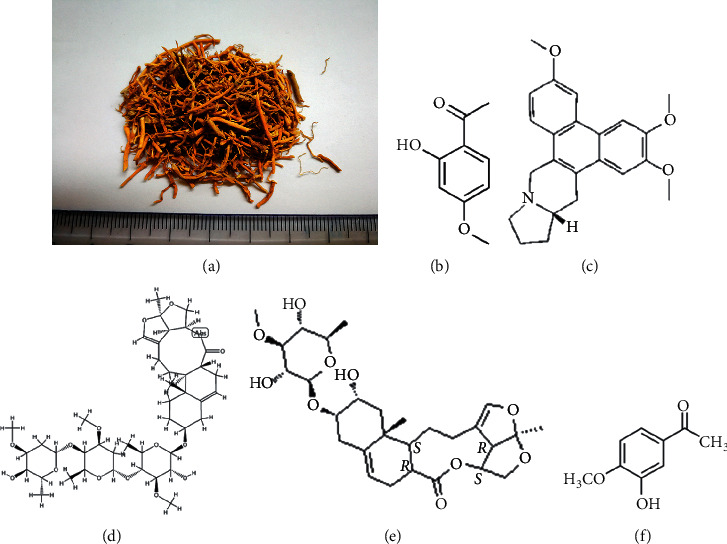
Dry herb of *Cynanchum paniculatum* and chemical composition of its major constituent: (a) dry herb of XCQ; (b) paeonol (C99H10O3, CAS number 552-41-0); (c) antofine (C23H25NO3, CAS number 32671-82-2); (d) cynanversicoside A (C42H64O15, CAS number 138875-31-7); (e) cynanversicoside C (C28H40O10, CAS number 934701-03-8); (f) 3-hydroxy-4-methoxy-acetophenone (C9H10O3, CAS number 6100-74-9).

*Cynanchum paniculatum* (*Xuchangqing* in Chinese, Figures 1(a)) has been used as folk medicine by Chinese medicine (CM) practitioners for more than two thousand years to treat various inflammatory diseases. The earliest record of *Cynanchum paniculatum* traced back to Shennong's Materia Medica Classic, which is the oldest Chinese pharmacopeia compiled in the *Han* Dynasty about 100 Anno Domini (AD). *Xuchangqing* is recognized as one of the top-grade drugs (tonic and nontoxic) and has a long history of clinical applications for treating infectious diseases [1]. As a folk medicine, the roots of *Cynanchum paniculatum* can be applied orally in the form of water extract either alone or in decoction. In CM theory, the main effects of *Xuchangqing* include dispelling wind, relieving pain, promoting blood circulation, detoxification, and reducing swelling [2, 3]. *Xuchangqing* is recognized in treating rheumatic arthralgia, odontalgia, dysentery, diarrhea, malaria, abdominal pain, chest pain, eczema, and urticaria. The use of *Xuchangqing* in managing these symptoms is linked to the traditional concept of treating wind-damp syndrome [2, 3].

Modern pharmacological studies show that the chemical composition of *Cynanchum paniculatum* majorly includes phenolic compounds [2, 3]. In recent years, the development of separation and detection techniques, such as liquid chromatography-mass spectrometry, facilitated the phytochemical studies on *Cynanchum paniculatum.* Researchers identified many secondary metabolites from *Cynanchum paniculatum*, including phenol derivatives, polysaccharides, glycosides, and alkaloids [4–7]. Pharmacological research shows that the chemical composition or extract of *Cynanchum paniculatum* has analgesic, sedative, anti-inflammatory, antibacterial, antiallergic, and anticancer effects [3, 8–10]. *Cynanchum paniculatum* is widely used for treating gastroenterological, oncological, dermatological, and orthopedics diseases in CM practices.

**Figure 2:**
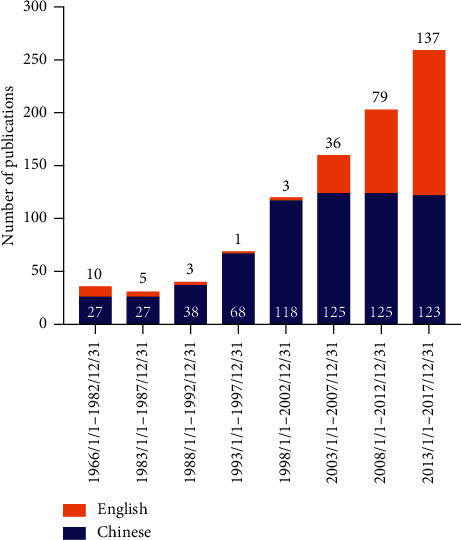
The number of publications by year showed an increase of research attention on *Cynanchum paniculatum* and its major constituents in recent years across the Chinese and English literature.

In the last decade, increasing research has focused on the *Cynanchum paniculatum* and its active components in the treatment of inflammatory diseases (Figure 2); however, a systematic review was not yet available. In this review, the advances in the traditional application, phytochemistry, pharmacology, and the current research progress of *Cynanchum paniculatum* on treating various diseases are systematically reviewed. Moreover, we focused on elaborating the potential molecular mechanism of the combination of *Cynanchum paniculatum* and other herbs, *in vivo* research, and clinical applications. The most relevant studies were published in English and Chinese. Electronic databases were extensively searched from their literature research through March 2020, using the following databases: US National Library of Medicine MEDLINE database (via PubMed), Allied and Complementary Medicine Database (AMED), Cumulative Index to Nursing and Allied Health Literature (CINAHL), and China Journals Full Text Database (via China National Knowledge Infrastructure, CNKI). The terms and keywords for searching included *Xuchangqing*, *Cynanchum paniculatum*, anti-inflammation, studies mechanism, pharmacology, toxicology, and *in vivo*. Further manual searches of our own documentation and cross-referencing of identified articles were conducted. After systematic review, we did not identify any previous review article regarding the anti-inflammatory effect, the application in inflammatory diseases, and safety *Cynanchum paniculatum* until the completion of this paper.

## 2. Traditional Usages of *Cynanchum paniculatum* in Chinese Medicine

In view of its wide range of pharmacological activities, *Cynanchum paniculatum* has long been used as a medicinal plant in China. Ever since the first documentation of *Xuchangqing* as a top-grade medicine in Shennong Materia Medica, *Cynanchum paniculatum* was frequently cited in many other medical literatures, such as the Supplement to Recipes Worth A Thousand Gold (Qianjin Yi Fang, *Tang* Dynasty, AD 682), Taiping Holy Prescriptions for Universal Relief (Tai Ping Sheng Hui Fang, *Song* Dynasty, AD 992), and Compendium of Materia Medica (Ben Cao Gang Mu, *Ming* Dynasty, AD 1590) for the treatment of wind-damp syndrome, similar to other acute infectious diseases such as tuberculosis, acquired immune deficiency syndrome (AIDS), malaria, fevers, acute urinary infection, and abscesses [11, 12]. However, there is a potential confusion on the source of *Xuchangqing* across ancient literature among *Dysosma versipellis*, *Dicliptera chinensis*, *Cynanchum atratum*, *Cynanchum stauntonii*, etc. [11, 13]. The reason for this confusion may be due to the use of *Cynanchum paniculatum* radix for medicinal purposes; the whole plant was not recorded in detail by ancient herbology literature [11, 13–15].

As a single herbal medication, *Xuchangqing* was documented in Shennong Materia Medica for the treatment of treating hallucination and mental disorder, parasitic and virus, phytophthora, and malaria [1]. It was recorded to promote health and general well-beings with long-term use [1]. In Simple Herbs (Jian Yi Cao Yao, *Qing* Dynasty, AD 1822), *Xuchangqing* was recorded to treat soft tissue injuries and pain [16], while in Medical Characteristics of Raw Herb (Sheng Cao Yao Xing Bei Yao, *Qing* Dynasty, AD 1872), *Xuchangqing* containing medicinal liquor was used for the treatment of rheumatoid arthritis [16]. As a component in prescriptions, contemporarily, *Xuchangqing* is recorded in Yangzheng Xiaoji capsules and Yunxiang Qufeng analgesic spray (Yun Xiang Qu Feng Zhi Tong Ting) in the 2015 edition Chinese Pharmacopoeia for the clinical application as adjuvant therapy for malignant diseases, aches, and pains of muscles and joints, respectively [17, 18].

Regarding the clinical application of *Xuchangqing* in daily CM clinics, our research group inherits the experience from a National Master of Traditional Chinese Medicine, Professor Xia Xiang, in our long-term clinical practice [19]. According to the recommended dosage by the pharmacoencyclopedia of Chinese medicinal herbs [20], *Xuchangqing* is commonly used at a conventional dose of 15 g–30 g per time into herbal decoctions. As a single herb, *Xuchangqing* water decoction at a dosage of 15 g per adult per day is commonly used to manage symptoms, such as nasal congestion caused by chronic rhinopharyngitis, runny nose and itching dry cough caused by chronic cough, eczema caused by allergic dermatitis, abdominal pain, and diarrhea caused by chronic enteritis in aims to regulate immune and inflammatory reactions. A dosage of 30 g per adult per day is commonly used to manage chronic joint pain and repeated tissue edema, such as rubella itching. As in drug pairs, the combination of *Xuchangqing* with *Jixuecao* (*Centella asiatica*) at 1 : 1 ratio is routinely used in clinical practices for management of liver fibrosis, interstitial lung disease, intestinal adhesion, and other fibrous hyperplasia caused by chronic inflammation. The combination of *Xuchangqing* with *Huangqi* (Astragali Radix) at 1 : 1 to 1 : 4 ratio is used to promote tissue repair for the treatment of recurrent oral ulcers, gastric erosion ulcers, vascular endothelial cell damage, etc. Furthermore, the combination of *Xuchangqing* with *Tufuling* (Smilacis Glabrae Rhizoma) at 1 : 1 ratio is used for the treatment of rheumatic arthritis, muscle diseases, and degenerative diseases of bone and joints. Together, the clinical application of *Xuchangqing* is closely related to its traditional CM effects of removing wind and pain, promoting blood circulation, clearing heat, and relieving itching. In combination with modern pharmacological research and molecular biology, understanding of inflammatory diseases will promote the accurate application and improve overall treatment efficacy.

## 3. Phytochemistry of *Cynanchum paniculatum*: A Focus on Phenolic Derivatives

The chemical composition of *Cynanchum paniculatum* is similar to the other Asclepiadaceae plants, which consists mostly of phenolic derivatives [21, 22]. There is increasing research and continuous identification of novel components of *Cynanchum paniculatum* with the advances in technologies regarding extraction, isolation, and identification. Paeonol (C99H10O3, CAS Number 552-41-0, PubChem CID 11092, Figure 1(b)) is a phenolic compound accounts for around 1% of the entire dry *Cynanchum paniculatum* and is also a major active compound in *Xuchangqing* [21, 22]. Other components, such as cynapanosides, phenanthroindolizidine alkaloid antofine (C23H25NO3, CAS Number 32671-82-2, PubChem CID 639288, Figure 1(c)), cynanversicoside A (C42H64O15, CAS Number 138875-31-7, Figure 1(d)) and cynanversicoside C (C28H40O10, CAS Number 934701-03-8, Figure 1(e)), and 3-hydroxy-4-methoxy-acetophenone (C9H10O3, CAS Number 6100-74-9, PubChem CID 24885509, Figure 1(f)) were identified as early as the 1980s to 1990s. Other components, such as neocynapanogenins and paniculatumosides, had also been identified [15, 21–23]. It is worth noting that in the recent five years, various new C21 steroidal glycosides have been discovered, such as paniculatumoside G and neocynapanogenin C identified in 2017 [24] and paniculatumoside H and paniculatumoside I identified in 2019 [25]. However, most of the biological activity assessments of these newly identified components remain preliminary at *in vitro* levels.

## 4. Anti-Inflammatory Pharmacological Studies of *Cynanchum paniculatum*

**Figure 3:**
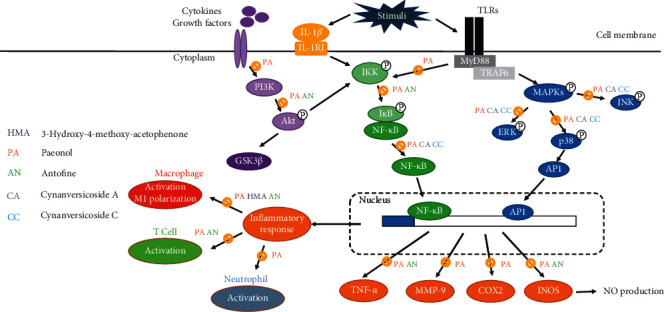
Schematic diagram of the anti-inflammatory pharmacological effects of *Cynanchum paniculatum*.

Inflammation is a complex biological response to living tissue injury by harmful stimuli, such as viral infection, microbial infection, chemical irritants, and damaged cells. The process of inflammation involved various aspects, including the immune cells, molecular mediators, and blood vessels. Aside from acute infection, where a specific pathogen triggers adaptive immunity, inflammation may also involve a variety of diseases, such as periodontitis, arthritis, asthma, gastrointestinal disorders, and even cancer. Several molecular mediators and pathways are closely connected with inflammatory responses, such as inflammatory cytokines tumor necrosis factor-alpha (TNF-*α*), interleukins (ILs), and cyclooxygenase- 2 (COX-2) [26, 27]. Furthermore, the nuclear factor-*κ*B (NF-*κ*B) pathway and mitogen-activated protein kinase (MAPK) signaling pathway are also of importance and are important targets of *Cynanchum paniculatum* and its derivatives. The mechanism is summarized in Figure 3.

### 4.1. Modulation of NF-*κ*B Pathway

NF-*κ*B represents a family of inducible transcription factors that can regulate various genes involved in immune and inflammatory responses [28]. The alteration of NF-*κ*B has contributed to the pathogenesis of many inflammatory diseases, such as inflammatory bowel disease, rheumatoid arthritis, chronic obstructive pulmonary disease (COPD), and asthma [29]. Inhibition of I*κ*B (inhibitor of NF-*κ*B) phosphorylation can subsequently inhibit NF-*κ*B translocation, thereby preventing NF-*κ*B subunits from binding to target genes and transcriptionally active inflammation. *Cynanchum paniculatum* had been demonstrated to dose-dependently suppresses muscle swelling by upregulating NF-*κ*B p65 subunit mRNA expressions, as well as modulate its downstream genes that mediate inflammatory mediators COX-2 and interleukin-1 beta (IL-1*β*) biosynthesis [30]. Furthermore, *Cynanchum paniculatum* and paeonol in pulmonary diseases, at least in part by the inhibition of Toll-like receptor 4 (TLR4)/NF-*κ*B inflammatory signaling [31, 32].

### 4.2. MAPK Signaling Pathway

In response to inflammatory stimuli, cytokines can act through Toll-like receptors and MAPK pathways leading to activation of NF-*κ*B. Three major groups of distinctly regulated mitogen-activated protein kinase cascades commonly altered inhuman diseases are extracellular signal-regulated kinases (ERK1/2), mitogen-activated protein kinase p38 (p38 MAPK), and c-Jun N-terminal kinase (JNK). Upon activation of the MAP kinases, transcription factors are phosphorylated and activated leading to a series of biological responses. In chronic bowel diseases, stroke, Alzheimer's disease, arthritis, and other chronic inflammation related diseases, MAPK pathways are frequently activated and identified as the inhibitor for drug development [33–35]. *Cynanchum paniculatum* and its derivatives have shown anti-inflammatory activities through suppressing lipopolysaccharides- (LPS-) induced inflammatory cytokines in macrophage as well as blocking MAPK/ERK/p38 signaling pathway [27, 36].

## 5. Immunomodulatory Effect of *Cynanchum paniculatum*

Recent studies have indicated that the anti-inflammatory effects of *Cynanchum paniculatum* and its derivatives are related to the modulation of the immune system, especially regulating the macrophages activation [10, 27, 36]. Antofine and its analogues could inhibit the LPS-induced nitric oxide (NO) production in RAW 264.7 macrophage cells [36, 37]. Antofine suppresses the expressions the inflammation related genes (such as ARG-1, IL1F9, IL-10, and IL-33), the extracellular matrix related genes (such as TNC and HYAL1), and the vasopressor gene (EDN1) in LPS-activated macrophage cells [37]. Paeonol suppresses inflammatory cytokines in LPS-induced macrophage cells and protects mice from lethal endotoxin shock by regulating the production of tumor necrosis factor-*α* (TNF-*α*), IL-1*β*, interleukin-6 (IL-6), and interleukin-10 (IL-10) via inactivation of I*κ*B*α*, ERK1/2, JNK, and p38 MAPK [27, 36]. Furthermore, paeonol also reduces the nucleocytoplasmic transportation of extracellular high mobility group box 1 (HMGB1) by upregulating histone deacetylase 3 (HDAC3) [38, 39]. It is worth noting that the anti-inflammatory and antioxidative activities of paeonol and its metabolites were found mostly through blocking MAPK/ERK/p38 signaling pathway and PI3K/Akt/NF-*κ*B pathway [27, 40, 41]. A recent study also suggests paeonol attenuates LPS-induced endothelial dysfunction and apoptosis by inhibiting bone morphogenetic protein 4 (BMP4) and TLR4 signaling independently [42]. Further study may provide further understanding to inflammatory disease and broadened the application of *Cynanchum paniculatum* and its derivatives.

## 6. Modulation of Inflammation by *Cynanchum paniculatum* and Its Components in Human Diseases

**Figure 4:**
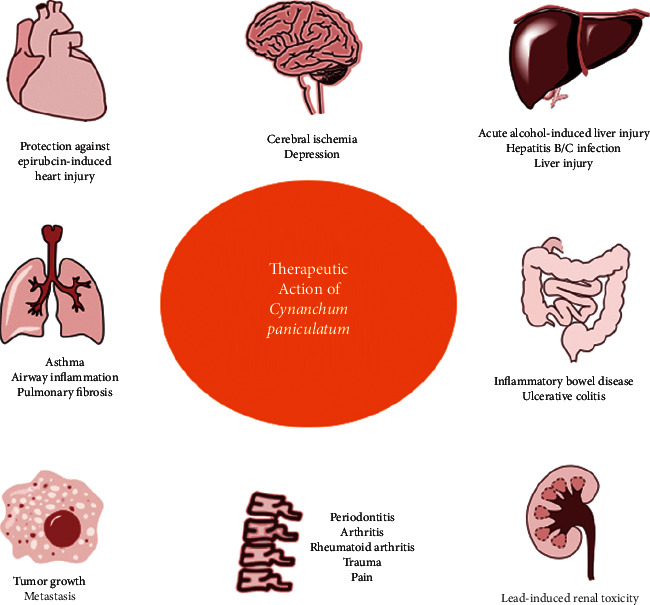
Role of *Cynanchum paniculatum* chronic inflammation related human diseases.

Chronic inflammation plays a major role in various chronic diseases, such as respiratory, cardiovascular, gastrointestinal, hepatobiliary, urogenital, neurological, skeletal, rheumatological systems, and cancer diseases [43–48]. The effect of *Cynanchum paniculatum* and its derivatives on inflammation-associated chronic human diseases are summarized by the systems, as shown in Figure 4 and Table 1.

### 6.1. Respiratory Diseases

As a folk medicine, *Cynanchum paniculatum* has a long history of application and a recognizable role for respiratory syndromes, such as cough, phlegm, and shortness of breath. Accumulating pharmacological evidence suggests the anti-inflammatory effects of *Cynanchum paniculatum* and its active constituents. In respiratory diseases, the inflammatory response can often be associated with an unfavourable prognosis. The elevation of inflammatory mediators, such as TNF-*α*, IL-1*β*, reactive oxygen species (ROS), and NO, can be cardiodepressive and can contribute to multiorgan dysfunction syndrome with disease progression. Recently, increasing research attention and research outcomes have suggested the role of *Cynanchum paniculatum* and paeonol in pulmonary diseases, including asthma, airway inflammation, cigarette smoke-induced pulmonary inflammation, and fibrosis [31, 49–52]. The mechanism of action mostly involves the inhibition of TLR4/NF-*κ*B and MAPK signaling and the inhibition of ROS-sensitive inflammatory signaling [31, 32]. These research outcomes provide, at least to a certain degree, a biomedical basis for the further investigation and application of *Cynanchum paniculatum* in the treatment of respiratory diseases, although few related mechanisms or targets were identified.

### 6.2. Cardiovascular Diseases

In *Shenghui Fang* (*Song* dynasty, 992 AD), *Cynanchum paniculatum* was documented for the treatment of chest pain, and ever since its documentation, *Cynanchum paniculatum* has been widely applied for the treatment of cardiovascular diseases. In recent years, the cardiovascular pharmacological effects of *Cynanchum paniculatum* and its active constituents aroused widespread interest among researchers. The possible mechanism of its action might include vascular dilation effect by modulating ion channels [53, 54], promoting oxidized low-density lipoprotein (LDL) uptake by macrophage, and regulating inflammation. Previous studies suggest that the cardioprotective effect of *Cynanchum paniculatum* is, at least in part, due to the vascular dilation effect of paeonol by an intracellular calcium regulatory mechanism [53, 54]. Furthermore, paeonol protects against epirubicin-induced heart injury via regulating miR-1 and PI3K/Akt pathway [55–57].

Atherosclerosis refers to the accumulation of fat, cholesterol, and other substances that led to vascular inflammation, promoting that the disease progression, when severe, can lead to coronary artery disease and heart attack. Paeonol has been shown to downregulate scavenger receptor-A (SR-A) expression, suppress uptake of oxidized LDL by macrophage, and inhibit macrophage-derived foam cell formation, which plays a crucial role in the occurrence and pathogenesis of atherosclerosis [58]. Studies have also shown that paeonol can reduce the levels of total cholesterol (TC), triglyceride (TG), and low-density lipoprotein cholesterol (LDL-C) to exert anti-atherosclerosis effects in murine models [59]. Moreover, paeonol reduces malondialdehyde-modified low-density lipoprotein (MDA-LDL) content, down-regulating NF-*κ*B expression. Additionally, paeonol intervention is closely related to the downregulation of other inflammation-related factors, including C-reactive protein (CRP), intercellular adhesion molecule 1(ICAM-1), vascular cell adhesion molecule 1 (VCAM-1), and monocyte chemoattractant protein-1(MCP-1) [60, 61]. Considerable documents are supporting the view that *Cynanchum paniculatum* can be a promising botanical remedy for cardiovascular diseases. However, current evidence is quite preliminary; the specific link between *Cynanchum paniculatum* and LDL scavenging still needs to be thoroughly investigated. Despite the potentially involved inflammation-related factors which had been listed in the publications, the corresponding pathways, genes, or cytokines that might be responsible for the activation of progression of atherosclerosis are barely understood.

### 6.3. Gastrointestinal Diseases

The treatment of the abdominal disorder is one of the major and earliest recorded effects of *Cynanchum paniculatum* as folk medicine and this action may be linked to the current disease concept including ulcer, inflammatory bowel disease, liver injuries, and ascites. A previous study found that *Cynanchum paniculatum* and its derivatives 3-hydroxy-4-methoxy-acetophenone and paeonol possess analgesic effect and gastrointestinal motility inhibitory action [8]. Interestingly, despite the poor water solubility of paeonol, the good oral absorption rate of paeonol may, at least in part, contribute to its dose-dependent therapeutic effect on ulcerative colitis *in vivo* [62].

Clinically, *Cynanchum paniculatum* containing herbal remedies have shown certain therapeutic effects in gastrointestinal diseases, such as gastritis, ulcerative colitis, and irritable bowel syndrome (IBS). Atrophic gastritis is a process of chronic inflammation of the gastric mucosa of the stomach leading to digestive problems and may develop into gastric cancer. Yang et al. reported a *Cynanchum paniculatum* containing herbal remedy, the Pingwei Xiaoyi Decoction, in treating 79 cases of patients with atrophic gastritis with gastric precancerous lesions identified by gastroscopy. After treatment with the decoction for 3–6 months, 96.2% of the patients reported symptom relief, and gastroscopy and pathological examination revealed an effective rate of 79.75% [63]. It is worth noting that, among the included patients, 24 cases presented with *Helicobacter pylori* infection. After treatment with the decoction, the negative conversion rate of *Helicobacter pylori* was 58.3%, indicating a certain therapeutic effect on *Helicobacter pylori* infection [63].

Moreover, the combination treatment of *Cynanchum paniculatum* and Radix Aucklandiae has been reported to reduce IBS-associated pain and diarrhea [64]. In a clinical observational study including 60 patients with IBS, *Cynanchum paniculatum* containing decoction, Li-chang-tang, relieves abdominal pain, diarrhea, and constipation in 75.9%, 78.4%, and 78.1%, respectively, after three weeks of treatment [65]. In an expert consensus published by the Chinese Society of Traditional Chinese Medicine Spleen and Stomach Disease Committee in 2009, for ulcerative colitis patients with abdominal pain, *Cynanchum paniculatum* is recommended to be included in treatment for pain relief [66]. Together, these studies suggest the nociceptive effects of *Cynanchum paniculatum* in gastrointestinal diseases. *Cynanchum paniculatum* might have an effect on inhibition of proinflammatory cytokine release, such as IL-1*β* and interferon-gamma (INF-*γ*), in digestive disorders. However, no experimental study is conducted to verify the role of *Cynanchum paniculatum* in cytokine release reaction. Furthermore, the clinical studies reported are mostly preliminary and observational and lack randomized control. Further investigations are needed to evaluate the clinical efficacy.

### 6.4. Hepatobiliary Diseases

The crucial roles of oxidative stress and inflammation in the development of the chronic liver disease have been emphasized for decades. Increasing evidence shows that inflammation is sustained and participated in the pathological process of chronic hepatitis, liver fibrosis, cirrhosis, and cancer. Approximately 25% of people with chronic hepatitis B viral (HBV) infection eventually develop cirrhosis or liver cancer [67]. Traditionally recognized as a detoxifying herb, *Cynanchum paniculatum* water extract showed a 50% inhibitory rate against HBV surface antigen (HBsAg) and e-antigen (HBeAg) at a dose of 0.78 g/L and 10.13 g/L, while the 50% cytotoxic dose toward hepatic cell is 62.65 g/L *in vitro* [68]. This experiment shows that *Cynanchum paniculatum* can effectively inhibit the secretion of two HBV antigens *in vitro* [68]. Other studies also suggested paeonol alleviated acute alcohol-induced liver injury via the SIRT1/Nrf2/NF-*κ*B signaling pathway [69] and has antioxidation, anti-inflammation, antiapoptosis, and autophagy-induction action *in vivo* [70–73]. Liver fibrosis is a twound-healing response of tissue to self-repairing after injury and hepatic stellate cells are recognized to play a key role in the initiation, progression, and regression of liver fibrosis. Studies have shown that paeonol can inhibit the proliferation of hepatic stellate cells [74] and promote their apoptosis, and its inhibitory activity is related to the inhibition of hepatic stellate cell collagen synthesis-related protein matrix metalloproteinase 1 (matrix metallopeptidase 1, MMP-1) and MMP-9 expression [75] as well as the TGF-*β*/Smad3 signaling [76]. Furthermore, paeonol attenuates oxidative stress and protects against acetaminophen-induced hepatotoxicity *in vivo* [77]. The low hepatotoxicity of the *Cynanchum paniculatum* and its protective role in liver injury allows its safe long-term use. However, clinical and laboratory studies of *Cynanchum paniculatum* and its derivatives in liver diseases are yet preliminary. The effects of *Cynanchum paniculatum* on oxidative stress and mitochondrial ROS generation, its scavenging ROS functions, and its antiapoptotic properties in chronic liver diseases are yet to be investigated. Considering the precise mechanism is still not clearly clarified, further studies focusing on the specific roles of *Cynanchum paniculatum* and its derivatives in chronic liver diseases should be emphasized.

### 6.5. Urogenital Diseases

In renal diseases, paeonol also possesses nephroprotective efficacy against lead-induced renal toxicity [78]. By suppressing endoplasmic reticulum (ER) stress in the kidney, paeonol reduces oxidative stress and decreases the NF-*κ*B activation and inflammatory cytokines TNF-*α* and IL-6 overproduction via the AMP-activated protein kinase (AMPK) and glycogen synthase kinase 3 (GSK-3) pathway *in vitro* and *in vivo* [78]. Similarly, in an epirubicin-induced renal injury mice model, paeonol has a protective effect on renal function by regulating the nuclear factor erythroid 2-related factor 2 (Nrf2) and NF-*κ*B pathways [56]. In a clinical study involving 30 patients with primary nephrotic syndrome treated with *Cynanchum paniculatum* containing remedy, the Yishen Jianpi decoction, it was reported that after 90 days of treatment, 16 out of 30 patients obtained complete relief from nephrotic syndrome. However, current studies lack investigation into precise mechanisms and clinical studies are yet observational; further studies are warranted.

In pelvic inflammatory disease (PID), *Cynanchum paniculatum* showed possible antimicrobial and anti-inflammatory activities as the principal component of xiaoyuningkun decoction, which also consists of *Melia toosendan* and *Angelica biserrata*. Xiaoyuningkun could significantly reduce the pain threshold in the mouse model of PID as well as the degree of inflammation in the uterus and Fallopian tubes compared with *Cynanchum paniculatum* decoction [79]. *Cynanchum paniculatum* decoction significantly reduced the serum levels of various inflammatory cytokines and adhesion molecules, including IL-1*β*, TNF-*α*, ICAM-1, and vascular endothelial growth factor (VEGF), and the expression of ICAM-1 and VEGF, in the mouse uterus and fallopian tubes [79]. These results suggest that *Cynanchum paniculatum* containing decoction had analgesic and anti-inflammatory effects and required further study.

### 6.6. Neurological Diseases

Neuroprotective effect of *Cynanchum paniculatum* may be linked to its traditional documentation of its invigorate blood, alleviate edema, and dredge collaterals effect. *Cynanchum paniculatum* methanol extract can protect mice hippocampal HT22 cells against glutamate-induced neuronal cytotoxicity [80, 81]. Consistently reported across various studies, paeonol can modulate NF-*κ*B signaling pathways, regulate MAPK signaling pathways, attenuate microglia-mediated inflammation, and oxidative stress-induced neurotoxicity both *in vivo* and *in vitro* [26, 82, 83]. Moreover, paeonol can suppress Toll-like receptor 2 (TLR2) and TLR4 signaling pathways and reduce proinflammatory factors in a cerebral ischemia-reperfusion injured rat model [84]. Furthermore, paeonol attenuates LPS-induced depressive-like behavior *in vivo* [85]. However, the exact action mechanisms are not yet fully elucidated.

### 6.7. Musculoskeletal Diseases

There is a long history of use of *Cynanchum paniculatum* in lower back pain, joint pain, and trauma. In fact, ancient Chinese documentation in the book of simplified herbs (*Jianyicaoyao*, in Chinese, *Qing* Dynasty) specifically stated the major action of *Cynanchum paniculatum* is for the treatment of trauma and joint pain. Contemporary studies found that paeonol alleviates IL-1*β*-induced osteoarthritis *in vivo* and alleviates pain and inflammation in murine complete Freund's adjuvant-induced arthritis model [86]. Furthermore, the antinociceptive effect of *Cynanchum paniculatum* has long been regarded to be related to its anti-inflammatory effect [3, 6, 8]. A previous study identified an antinociceptive effect of the ethyl acetate fraction of *Cynanchum paniculatum* via oral administrations in the inflammatory pain model *in vivo* [3]. In daily CM practices, the combined formula in the form of water decoction is commonly used in aims to enhance therapeutic efficacy; however, the fractions of the bombinated herbal mixture of *Cynanchum paniculatum* were not yet studied in detail [3]. A study investigated the various fractions of an anodyne spray (XQAS) containing ethanol extracts of two herbs, *Cynanchum paniculatum* and *Illicium henryi* with topical administration for acute soft tissue injury *in vivo* [30]. The results showed both short-term and long-term analgesic effects of XQAS [30]. Upon administration, XQAS rapidly suppresses inflammatory mediators such as prostaglandin-E2 (PGE2), IL-1*β*, and NO [30]. Further, XQAS long-term dose-dependently suppresses muscle swelling by upregulating NF-*κ*B p65 mRNA expressions, as well as modulating its downstream genes that mediate inflammatory mediators COX-2 and IL-1*β* biosynthesis [30].

*Cynanchum paniculatum* has long been recorded for the treatment of tooth pain in Chinese literature and several clinical observations have demonstrated its potential efficacy [87]. Mechanism wise, in a ligation-induced periodontitis rat model, paeonol attenuates by inhibiting osteoclastogenesis via regulating Nrf2/NF-*κ*B/NFATc1 signaling pathway [88], while, *in vivo*, intraperitoneal injection of paeonol reduced the induced osteoclast formation and possessed a consistent antidestructive effect in rat experimental periodontitis models [87, 88]. These results suggested a potential anti-inflammatory effect of paeonol on gingival tissue and the preventive role in alveolar bone loss during the process of periodontitis [87, 88]. These studies provided a scientific basis that proinflammatory cytokines inhibition may, at least in part, contribute to the treatment effect of *Cynanchum paniculatum* as folk medicine for periodontitis. Despite different models involved in these experiments, the role of *Cynanchum paniculatum* in arthritis and periodontitis requires further exploration. Besides, there are no clear and standard therapeutic dosages of *Cynanchum paniculatum* in studying the nociceptive effect of *Cynanchum paniculatum* across disease models.

### 6.8. Rheumatological and Dermatological Diseases

#### 6.8.1. Rheumatological Diseases

In rheumatoid arthritis, paeonol possesses protective effects on inflammatory response in IL-1*β*-induced human fibroblast-like synoviocytes during disease progression via modulating the NF-*κ*B pathway [89, 90]. Furthermore, paeonol, by upregulating forkhead box O3 (FOXO3) through inhibition of miR-155 expression, protects against TNF-*α*-induced proliferation and cytokine release of rheumatoid arthritis fibroblast-like synoviocytes [89, 90]. *Cynanchum paniculatum* containing herbal capsule, the compound Fengshiding capsule (CFC), which also contains *Alangium platanifolium*, *Angelicae dahurica*, and *Glycyrrhiza uralensis* has been widely used as a clinical therapy against rheumatoid arthritis. Adopting the network pharmacology scheme, the potential pharmacological targets of CFC components in rheumatoid arthritis were investigated by Duan et al. [91]. It was found that CFC alters metabolic and immune-related pathways and possesses apoptotic, oxidative stress modulatory and anti-inflammatory effects that cumulatively serve for its clinical application against rheumatoid arthritis. These results supported the protective effect of *Cynanchum paniculatum* and its derivative on rheumatological disease progression and the mechanism might be related to inhibiting overexpressed levels of inflammatory cytokines. However, direct pathways involved in regulating hepcidin need to be investigated in the future.

#### 6.8.2. Dermatological Diseases

Various clinical observations have reported the treatment effect of *Cynanchum paniculatum* in dermatological diseases. *In vivo* and *in vitro* studies suggested a protective role of paeonol against solar ultraviolet- (SUV-) induced skin inflammation via targeting T-LAK cell-originated protein kinase (TOPK), an upstream of p38 kinases and JNKs [92]. Furthermore, paeonol inhibits IL-6 and TNF-*α* secretion in a skin inflammation mouse model [93]. Paeonol ameliorates imiquimod-induced psoriasis-like skin lesions by inhibiting the maturation and activation of dendritic cells *in vivo* [93]. Other clinical observational studies suggest that *Cynanchum paniculatum* possesses antipruritic effects in recurrent urticaria, tinea dermatosis, or pityriasis when used in decoction or external application as washing liquid [94, 95]. It is also worth highlighting that *Cynanchum paniculatum* containing topical ointment can alleviate skin vasculitis and is a cost-effective treatment option [96].

### 6.9. Malignant Diseases

The relationship between inflammation and cancer has become a hot topic for cancer research in recent years. During the process of inflammation, cytokine and growth factors are produced and subsequently activate downstream cancer-related gene and transcription factors, including NF-*κ*B [97–99]. Although there is an absence of the concept of cancer in ancient CM, some CM syndromes, such as damp-wind or toxicity (also known as *Feng, Shi, Du* in Chinese), can be easily linked to the modern concept of infection or inflammation. In view of CM, the potential anticancer effect of *Cynanchum paniculatum* can be ascribed to one of the CM traits by clearing wind-damp or removing toxicity. Despite the lack of direct claims to the anticancer properties of *Cynanchum paniculatum*, the link between anticancer and anti-inflammation can be established after anti-inflammation, anticachectic, antioxidant, antiproliferation, and antiinvasion properties [99–103].

*Cynanchum paniculatum* and/or its formulae have shown anticancer potential discovered *in vitro* and *in vivo* [9, 21, 55, 104]. Several studies have identified that *Cynanchum paniculatum* and its major components could interfere with inflammatory signaling and thereby suppresses tumor development, growth, progression, and metastasis. For example, paeonol and antofine extracted from *Cynanchum paniculatum* have an inhibitory effect on tumor cell growth [21, 100, 105] dose- and time-dependently [21, 98, 100, 105, 106]. In diethylnitrosamine-induced hepatocellular carcinoma rat models, paeonol significantly improves immunity function, protects against oxidative injury, and improves liver function [107]. It is reported that paeonol can induce apoptosis by affecting the expression of apoptosis-related genes [108–111] and has antitumor effects *in vitro* and *in vivo* [100, 103], and is selective for different cell lines.

Our research group previously identified that paeonol could inhibit epithelial-mesenchymal transition of pancreatic adenocarcinoma *in vitro* via suppressing the transforming growth factor beta 1(TGF-*β*1)/SMAD signaling pathway provided some evidence for future investigation into the antimetastasis effect of *Cynanchum paniculatum* and its derivatives [112]. Although current literature suggests an anticancer progression and metastasis role of *Cynanchum paniculatum* and its derivatives, no clinical experimental study has been conducted to verify the role of *Cynanchum paniculatum* either use as a single agent or in decoction as complementary and alternative medicine in cancer patients. Furthermore, the potential effect and interaction of *Cynanchum paniculatum* with chemotherapy, immunotherapy, and target therapy need to be carefully investigated in the future concerning both the efficacy and safety of the future clinical applications.

## 7. Safety and Quality Control of *Cynanchum paniculatum*

The 50% lethal dose (LD50) of mice injected into the peony phenol preparation by intraperitoneal injection was 32.9 ± 1.0 g/kg [113]. When the rabbit is injected intravenously at 5 g/kg, convulsions can occur for 30–60 seconds. The animal is in good condition within 48 hours [113]. It is worth noting that according to the pharmacoencyclopedia of Chinese medicinal herbs, the quality control of *Xuchangqing* should contain no less than 1.3% of paeonol on high-performance liquid chromatography (HPLC) fingerprint of Radix *Cynanchum paniculatum* [20]. Several studies have suggested that the amount of paeonol in *Cynanchum paniculatum* was affected by the culture environment and extraction method [20, 114]. The dried raw herb *Xuchangqing* shall also be followed by other requirements (e.g., water content should not more than 16.0% and foreign matter should not more than 6.0%, etc.) according to the Chinese pharmacopoeia [20]. Alarmingly, in a large, cross section survey study of pesticide contamination among 1771 herbal medical samples from 503 major cultivation areas, *Cynanchum paniculatum* was investigated among them [115]. The pesticide analysis result showed that 19 pesticides were detected in which seven of them were banned while eight are overlimited. The result strongly indicated that obtaining a pesticide contamination report before introducing *Cynanchum paniculatum* into clinical usage is of great importance to avoid pesticide-related risk.

Among the *Cynanchum* genus, there are some important medicinal species in China, such as *Cynanchum paniculatum* and *Cynanchum atratum* [13, 114]. The medicinal species in Cynanchum are easily confused, leading to potential safety risks. Through the development of identification technology, researchers identified *Cynanchi Atrati* Radix et Rhizoma (*Baiwei* in Chinese) showed a potential safety problem by discriminating 64 commercial samples of the medicinal plants [116]. By employing the internal transcribed spacer 2 (ITS2) barcode, researchers surveyed the authentication and greatly improved the identification efficiency and accuracy of commercial herbal medicines in the *Cynanchum* genus [13, 114, 116]. In 2006, Deng et al. reported quick and easy identification of paeonol content by the microwave-assisted extraction and headspace single-drop microextraction (MAE-HS-SDME) method for quality monitoring for the two herbal medications of *Cynanchum paniculatum* and *Paeonia suffruticosa* [13, 114, 117]. The further development of quantitative and qualitative determination of major chemical composition is warranted to improve the quality control of *Cynanchum paniculatum* for providing safer pharmacological applications.

## 8. Discussion

In recent years, significant achievements have been made on the identification of active components of *Cynanchum paniculatum* and the related action of targets in infectious diseases and chronic inflammatory diseases. In this review, the anti-inflammatory effect of *Cynanchum paniculatum* is linked with its ancient traditional application. Mechanism studies revealed that interference with NK-*κ*B and MAPK pathways are major contributors to the immunomodulation effect, the anti-inflammatory effect, and the antioxidant effects of *Cynanchum paniculatum* and its bioactive components.

Through the literature review, most studies involved the canonical NF-*κ*B pathway. The noncanonical NF-*κ*B pathway in response to stimuli including the TNF receptor superfamily (TNFRSF), such as B cell-activating factor receptor (BAFF-R), cluster of differentiation 40 (CD40), and receptor activator of nuclear factor kappa-Β ligand (RANKL), are worthy of further investigation to clarify the role of *Cynanchum paniculatum* in NF-*κ*B-inducing kinase, p100 phosphorylation, and nuclear translocation of noncanonical NF-*κ*B complex p52/RelB. In addition, the NF-*κ*B pathway can regulate the activation, differentiation, and effector function of inflammatory T cells. Furthermore, NF-*κ*B is a central mediatory or NLRP3 inflammasome activation and pro-IL-1*β* response and may serve as an autoregulatory mechanism to regulate proinflammatory function. Further investigation into the related pathways may be promising for further understanding of the action mechanism of the anti-inflammatory effects of *Cynanchum paniculatum* and aids in the elucidation and understanding of chronic inflammatory diseases.

Recently, increasing attention has been drawn on the energy metabolic homeostasis and chronic inflammation. Metabolic syndrome, for example, had been associated with a low-grade, chronic inflammation resulting from chronic stress from nutritional overload. Paeonol has been suggested as anti-inflammatory via the AMPK pathway, where emerging results indicated AMPK signaling could inhibit the inflammatory response by NF-*κ*B pathways. Instead of directly phosphorylated NF-*κ*B subunits, AMPK signaling can modulate several downstream targets, such as SIRT1, PGC-1*α*, p53, and FoxO factors that subsequently inhibit NF-*κ*B signaling. This aspect is also worthy of further investigation. Together, the promising anti-inflammatory roles of *Cynanchum paniculatum* provide scientific support for future clinical application, and other identified compounds isolated from *Cynanchum paniculatum* may be promising, such as antofine, cynanversicoside A, and cynanversicoside C and requires further study. Moreover, further investigation into a new mode of herbal medicine based on the “composition structure theory” [118] according to different functional units and chemical constituents may provide novel insights and broaden the future application of *Cynanchum paniculatum*. Furthermore, in in depth study into the pharmacological mechanism, pharmacokinetic and pharmacodynamics are warranted for better understanding of the role of Cynanchum paniculatum in inflammatory-related diseases.

Our research group has been studying the pathogenesis of atherosclerosis and its treatment with herbal medication [119]. Modern biomedical studies suggest that atherosclerosis is an intravascular chronic inflammation. Our preliminary clinical observation identified that herbal medication with replenishing *Qi* and activating blood circulation effects under CM theory possess anti-inflammatory and hypolipidemic effects by reducing arterial vessel wall thickening in patients with femoral atherosclerosis. In the past 20 years, a *Xuchangqing* containing CM decoction “*Xu-Huang* Mixture,” which contains *Astragali Radix* (*Huangqi*), *Typhae Pollen* (*Puhuang*), and *Epimedium brevicornum* Maxim (*Xianlingpi*) is long used for the treatment of coronary heart disease in our group. Our preliminary clinical observational study showed an improvement of cardiac function and reversal or arterial plaque in patients with coronary atherosclerotic diseases when treated with *Xu-Huang* mixture and standard care. We further investigated the role of *Xu-Huang* mixture in patients with diabetic lower extremity atherosclerotic occlusive disease. Our results showed a significant improvement of anterior tibial artery intima-media thickness (IMT), posterior tibial artery IMT, dorsalis pedis artery IMT, blood viscosity, fibrinogen, plasma D-dimer, total cholesterol, low-density lipoprotein cholesterol, and high-density lipoprotein cholesterol (*P* < 0.05). Furthermore, Xu-Huang mixture is safe without obvious hepatic or renal toxicities. CM herbs have been characterized as multi-ingredient, multitarget therapies with low adverse effects, making them advantageous in treating certain chronic diseases. A herbal decoction is a complex mixture of different, but often closely related, herbs and compounds. The form of decoction is relatively low in toxicity due to the generally low concentration of each of the constituents, and the synergism between different constituents makes herbal decoction an interesting “cocktail” of the natural origin that can act on different targets. Further randomized double-blind clinical trials of *Xu-Huang* mixture for diabetic lower extremity atherosclerotic occlusive disease are needed and its expanded application to other vascular inflammatory lesions is worthy of further instigations.

## 9. Conclusion

All in all, most of the studies regarding the anti-inflammatory property of *Cynanchum paniculatum* and its major active constituents were recently conducted and identified in the past ten years. Despite the long history of clinical application of *Cynanchum paniculatum* in CM practice, many more details of its clinical potential, such as the role in inflammatory vascular diseases, future use as anticancer molecular targets, its indication on different individuals, and its pharmacodynamics and pharmacokinetics remain uncovered. Prospectively, there is a need for *in vivo* studies to ascertain the effect of *Cynanchum paniculatum* extract and its major active constituents via various application routes, such as topical, oral administration, or in the form of injections. The prospective uses of *Cynanchum paniculatum* in the treatment of inflammatory diseases can be derived from both traditional knowledge and modern basic research. Future the application of genomics and proteomics approaches, as well as computer-aided molecular design, may unveil novel therapeutic targets and facilitate the understanding of inflammatory diseases. In conclusion, the anti-inflammation role of *Cynanchum paniculatum* and its bioactive components are of the therapeutic potential and worth further studies.

## Figures and Tables

**Table 1:** Pharmacological actions of *Cynanchum paniculatum* and its major active constituents in inflammatory-related diseases.

System of diseases	Name of diseases	Related targets and pathways	References
Respiratory	Asthma	IFN-*γ*↑, IL-4↓, IL-13↓, TLR4/NF-*κ*B signaling, MAPK signaling	[32, 49]
	Lung acute injury	IL-6↓, MCP-1↓, HMGB1↓, NF-*κ*B p65↓, TNF-*α*↓	[50, 52]
	Cigarette smoke-induced pulmonary inflammation	MAPKs/NF-*κ*B signaling, IL-8↓, ROS↓	[31]
	Lung fibrosis	TGF-*β*1-induced MAPKs/Smad3 signaling	[51]

Cardiovascular	Epirubicin-induced heart injury	miR-1↓, PI3K/Akt/mTOR pathway, NF-*κ*B signaling	[55]
	Atherosclerosis	SR-A↓, TC↓, TG↓, LCL-C↓, MDA-LDL↓, CRP↓, ICAM-1↓, VCAM-1↓, MCP-1↓	[58–61]
	Chronic gastritis	Clinical, *Helicobacter pylori*↓	[63]
	Irritable bowel syndrome	Clinical, pain↓	[65, 66]

Hepatobiliary	Chronic hepatitis B	HBsAg↓, HBeAg↓	[68]
	Acute alcohol-induced liver injury	SIRT1/Nrf2/NF-*κ*B signaling	[69]
	Acetaminophen-induced hepatotoxicity	Antioxidative, ALT↓, AST↓, p-JNK↓, TNF-*α*↓, MCP-1↓, IL-1*β*↓, IL-6↓, NF-*κ*B signaling pathway	[77]
	Liver fibrosis	Hepatic stellate cells↓, NF-*κ*B signaling, TGF-*β*/Smad3 signaling, MMP-1↓, MMP-9↓	[74–76]

Urogenital	Renal injury and renal toxicity	ER stress↓, oxidative stress↓, NF-*κ*B signaling↓, AMPK signaling↓, GSK-3 signaling pathway↓, IL-1*β*↓, TNF-*α*↓, ICAM-1↓, VEGF↓, Nrf2↑, HO-1↑	[56, 78]
	Chronic pelvic inflammation	IL-1*β*↓, TNF-*α*↓, ICAM-1↓, VEGF↓	[56]

Neurological	Neuronal cytotoxicity	NF-*κ*B signaling↓, MAPK signaling↓	[26, 80–83]
	Cerebral ischemia-reperfusion injury	TLR2↓, TLR4↓	[84]

Musculoskeletal	Osteoarthritis, soft tissue injury, trauma	PGE2↓, IL-1*β*↓, NO↓, NF-*κ*B signaling↓	[3, 6, 8, 30, 86]
	Periodontitis	Nrf2/NF-*κ*B/NFATc1 signaling	[87, 88]

Rheumatological	Rheumatoid arthritis	FOXO3↑, miR-155↓, NF-*κ*B signaling	[89, 90]

Dermatological	Solar ultraviolet-induced skin inflammation	TOPK↓, p-p38↓, JNKs↓, MSK1↓, histone H2AX↓, IL-6↓, TNF-*α*	[92]
	Psoriasis	MyD88↓, TLR8↓	[93]
	Dermatosis	Clinical	[95]
	Eczema	Clinical	[94]

Malignant	Breast cancer	CXCL4/CXCR3-B signaling, apoptosis↑	[103]
	Colon cancer	RUNX3↑, intracellular Ca^2+^↑, apoptosis↑, PGE2↓, COX-2↓	[98, 106]
	Esophageal cancer	Bcl-2/Bax↓, apoptosis↑	[108, 109]
	Gastric cancer	ERBB2↓, NF-*κ*B signaling, MMP-2↓, MMP-9↓	[97, 100, 102]
	Liver cancer	NF-*κ*B signaling	[107]
	Melanoma	TNF-*α*-activated NF-*κ*B and IL-6-activated STAT3 signaling	[99, 101]
	Ovarian cancer	VEGF↓, HIF-1*α*↓, PI3K/Akt pathway	[105, 110]
	Pancreatic cancer	TGF-*β*1/SMAD signaling	[112]
	Prostate cancer	Apoptosis↑, caspase-3↑, −8↑, −9↑, PI3K/Akt pathway	[111]

## Abbreviations

CM: Chinese medicine
AMED: Allied and Complementary Medicine Database
CINAHL: Cumulative Index to Nursing and Allied Health Literature
CNKI: China National Knowledge Infrastructure
TNF-*α*: Tumor necrosis factor-alpha
ILs: Interleukins
COX-2: Cyclooxygenase-2
NF-*κ*B: Nuclear factor-*κ*B
MAPK: Mitogen-activated protein kinase
COPD: Chronic obstructive pulmonary disease
IL-1*β*: Interleukin-1 beta
TLR4: Toll-like receptor 4
ERK1/2: Extracellular signal-regulated kinases
JNK: c-Jun N-terminal kinase
LPS: Lipopolysaccharide
IL-6: Interleukin-6
IL-10: Interleukin-10
NO: Nitric oxide
ARG-1: Arginase 1
IL1F9: Interleukin-1 family member 9
IL-33: Interleukin-33
TNC: Tenascin C
HYAL1: Hyaluronoglucosaminidase 1
EDN1: Endothelin 1
HMGB1: High mobility group box 1
HDAC3: Histone deacetylase 3
PI3K: Phosphoinositide 3-kinases
Akt: Protein kinase B
BMP4: Bone morphogenetic protein 4
ROS: Reactive oxygen species
LDL: Low-density lipoprotein
SR-A: Scavenger receptor-A
TC: Cholesterol
TG: Triglyceride
LDL-C: Low-density lipoprotein cholesterol
MDA-LDL: Malondialdehyde-modified low-density lipoprotein
CRP: C-reactive protein
ICAM-1: Intercellular adhesion molecule 1
VCAM-1: Vascular cell adhesion molecule 1
MCP-1: Monocyte chemoattractant protein-1
IBS: Irritable bowel syndrome
INF-*γ*: Interferon-gamma
HBV: Hepatitis B virus
HBsAg: Hepatitis B surface antigen
HBeAg: Hepatitis B e-antigen
SIRT1: NAD-dependent protein deacetylase sirtuin-1
Nrf2: Nuclear factor erythroid 2-related factor 2
MMP-1: Matrix metallopeptidase 1
MMP-9: Matrix metallopeptidase 9
ER: Endoplasmic reticulum
AMPK: AMP-activated protein kinase
GSK-3: Glycogen synthase kinase 3
PID: Pelvic inflammatory disease
VEGF: Vascular endothelial growth factor
TLR2: Toll-like receptor 2
XQAS: Xiangqing anodyne spray
PGE2: Prostaglandin-E2
NFATc1: Nuclear factor of activated T cells 1
FOXO3: Forkhead box O3
CFC: Compound Fengshiding capsule
SUV: Solar ultraviolet
TOPK: T-LAK cell-originated protein kinase
TGF-*β*1: Transforming growth factor beta 1
HPLC: High-performance liquid chromatography
MAE-HS-SDME: Microwave-assisted extraction and headspace single-drop microextraction
TNFRSF: Tumor necrosis factor receptor superfamily
BAFF-R: B cell-activating factor receptor
CD40: Cluster of differentiation 40
RANKL: Receptor activator of nuclear factor kappa-Β ligand
NLRP3: NOD-, LRR-, and pyrin domain-containing protein 3
PGC-1*α*: Peroxisome proliferator-activated receptor-gamma coactivator 1 alpha
IMT: Intima-media thickness.
